# Betaine supplementation affects carbohydrate metabolism in the skeletal muscle of finishing pigs

**DOI:** 10.1371/journal.pone.0322040

**Published:** 2025-04-29

**Authors:** Gustavo de Amorim Rodrigues, Alysson Saraiva, Marcos Henrique Soares, Tiago Antônio Oliveira Mendes, Thais Correa Costa, Caroline Brito da Silva, Pedro Vieira Monteiro, Marcio Duarte

**Affiliations:** 1 Department of Animal Science, Universidade Federal de Viçosa, Viçosa, Minas Gerais, Brazil; 2 Muscle Biology and Nutrigenomics Laboratory, Universidade Federal de Viçosa, Viçosa, Minas Gerais, Brazil; 3 Department of Animal Science, Universidade Federal de Minas Gerais, Belo Horizonte, Minas Gerais, Brazil; 4 Department of Biochemistry and Molecular Biology, Universidade Federal de Viçosa, Viçosa, MG, Brazil; 5 Department of Animal Biosciences, University of Guelph, Guelph, Ontario, Canada; University of Illinois, UNITED STATES OF AMERICA

## Abstract

This study aimed to assess the effect of betaine supplementation on the proteomic profile of the *longissimus thoracis* muscle in finishing pigs. Thirty-six crossbred female pigs (initial body weight = 88.96 ± 3.48 kg) were allocated in a completely randomized experimental design with two dietary treatments, with nine replications per treatment and two pigs per replication. The experimental diets consisted of a control diet (CON) and the CON diet supplemented with 2.5 g/kg of betaine (BET). At the end of the trial, one pig per unit was slaughtered, and *longissimus thoracis* (LT) muscle samples were analyzed via mass spectrometry to identify differentially abundant proteins (DAPs). Additionally, the intramuscular fat (IMF) content in the LT muscle was evaluated. Network analyses were performed to identify the biological processes and KEGG pathways. Results indicated that eleven DAPs were down-regulated (*q*-Value < 0.05) in the BET group compared to the CON group. Most DAPs were associated with carbohydrate metabolism, indicating that betaine may modulate cellular energy metabolism. These proteins were involved in glycolysis, glycogenolysis, the pentose phosphate pathway, and the glucagon signaling pathway. Moreover, pigs in the BET group had higher (P = 0.02) IMF compared to the CON group. In conclusion, betaine supplementation in finishing pigs down-regulated proteins related to carbohydrate metabolism in skeletal muscle, suppressed glucose metabolic processes and carbohydrate catabolic processes. These findings indicate alterations in glucose flow, possibly favoring other metabolic pathways, such as the pentose phosphate pathway. Furthermore, betaine supplementation increased IMF deposition and improved meat tenderness.

## 1. Introduction

As production costs and environmental concerns continue to rise steadily, enhancing growth performance, focusing on feed efficiency, is essential to ensure sustainable and competitive pig production. Particularly during the finishing phase, a decrease in body weight gain efficiency of pigs occurs [1]. This decline is due to increased fat deposition, along with a slowdown in the rate of muscle growth [1,2]. This change in tissue deposition becomes even more evident in pigs slaughtered at higher weights, which nowadays has become a trend but can compromise carcass quality and contribute to increased production costs [2]. Given this scenario, the inclusion of feed additives in the diet, such as betaine, which has been shown to stimulate skeletal muscle growth and reduce subcutaneous fat, especially in pigs with high genetic potential for muscle growth, has been recognized as a potential strategy to maximize feed efficiency [3–5].

Betaine, also known as trimethylglycine, plays an essential role as a methyl group donor, contributing to the synthesis of metabolically active compounds [6,7]. Pioneering research conducted by Cadogan et al. [8] and Feng [9] highlighted the beneficial effects of betaine, such as reducing subcutaneous fat thickness and increasing intramuscular fat (IMF) accumulation in finishing pigs, prompting growing interest in the use of betaine to regulate carcass fat composition. Since then, studies using targeted approaches, such as mRNA expression analysis, have suggested molecular mechanisms by which betaine may influence energy partitioning, especially in lipid metabolism regulation [10–13].

In skeletal muscle tissue, betaine supplementation is associated with increased de novo lipogenesis and reduced lipolysis, resulting in increased IMF deposition, which is a key determinant of meat quality [13,14]. The increase in IMF correlates positively with meat tenderness, significantly contributing to the improvement of sensory quality, while also meeting the growing demand from consumers for high-quality and nutritious meat products [15,16]. However, the underlying mechanisms of these molecular processes have not been clearly established, as they are complex and involve the regulation of multiple genes, both coding and non-coding [4,5,17]. Additionally, they are influenced by various factors, including carbohydrate metabolism, in which glucose availability and the activity of glycolytic enzymes play crucial roles in the *de novo* synthesis of fatty acids and triglycerides [18,19]. Therefore, the adoption of quantitative analysis techniques, such as proteomics, proves advantageous, enabling detailed characterization of the pig skeletal muscle proteome. This not only allows the identification of functional proteins but also establishes their interactions and enhances understanding of enriched molecular pathways [20,21].

Based on function and solubility, muscle proteins are divided into three main groups: sarcoplasmic, myofibrillar, and structural proteins [21,22]. The sarcoplasmic fraction consists of soluble proteins and enzymes, which play a fundamental role in the biochemical processes that influence overall muscle energy metabolism [23,24,25]. In this context, we hypothesize that betaine supplementation in finishing pigs influences the proteome of skeletal muscle, resulting in alterations in sarcoplasmic proteins. This study is the first to investigate, through proteomic analysis, how betaine supplementation may influence the sarcoplasmic proteome of skeletal muscle in finishing pigs. Thus, the aim of this study was to analyze the effect of betaine supplementation on the proteomic profile of the *longissimus thoracis* muscle in finishing pigs.

## 2. Materials and methods

All methods involving the handling of pigs followed the ethical principles of animal research (CONCEA) and were approved by the Commission of Ethics in the Use of Production Animals (CEUAP) of the Universidade Federal de Viçosa (protocol 013/2018).

### 2.1. Animals, experimental design and tissue sampling

Thirty-six commercial crossbred female pigs with an average initial weight of 88.96 ± 3.48 kg were obtained from the same source and were allocated in a completely randomized experimental design with two dietary treatments with nine replications, and two pigs per experimental unit (represented by the pen). All pigs in this study were selected from a single barn and were similar in their husbandry practices and genetic background [PIC 337 (Large White × Landrace × Duroc × Pietrain) × Camborough (Large White × Landrace)]. The experimental diets consisted of a control diet (CON) mainly composed of corn, soybean meal, amino acids, and supplements of minerals, and vitamins [13] to meet the nutrient requirements of finishing pigs [26]; or the CON diet supplemented with 2.5 g/kg of betaine (BET; Betaine 72.5% purity, Shandong Aocter Chemical Co., Ltd., Liaocheng, China) in replacement of inert clay filler. The pigs were housed in pens (2.30 × 2.10 m, 4.83 m² total, 2.415 m² per pig) with concrete-floored and masonry walls, equipped with a semi-automatic feeder and a nipple drinker, and had free access to feed and water throughout the 45-d feeding trial.

On the 45th day of the feeding trial, all pigs were weighed, and the pig closest to 140 kg within each experimental unit was selected from each pen. Subsequently, the pigs underwent a 14-hour fasting period, with free access to water. Following this period, the selected pig was electrically stunned and slaughtered to collect samples from the *longissimus thoracis* muscle (n=18, with nine pigs per dietary group). Immediately after slaughter, the area between the 12th -13th rib on the left side of the carcass was initially cleaned with 70% ethanol, and the incision was performed made to collect samples from the *longissimus thoracis* muscle (~20 g). Right after collection, the samples were rinsed with phosphate saline buffer (pH = 7.4), placed in a 2-mL cryogenic vial (Corning, USA) and snap-frozen in liquid nitrogen and stored at −80 °C.

After slaughter, the carcasses were chilled at 5°C for twenty-four hours. Subsequently, the sides of the carcasses were ribbed at the 10th rib location, and the backfat thickness over the *longissimus thoracis* muscle, 6 cm from the midline, was measured using a digital caliper. Then, approximately 20 cm samples of the *longissimus thoracis* muscle were collected from the right side of the carcass, between the 10th rib and the first lumbar vertebra, for meat quality analysis. The samples were then sliced into chops (~ 3 cm thick) and stored in a freezer at -20°C.

After slaughter, the carcasses were chilled at 5°C for twenty-four hours, and then, approximately 20 cm samples of the *longissimus thoracis* muscle were collected from the right side of the carcass, between the tenth rib and the first lumbar vertebra, for meat quality analysis. The samples were sectioned into chops (~ 3 cm in thickness) and stored in a freezer at -20°C.

### 2.2. Extraction of sarcoplasmic proteins and LC-MS/MS

The method for extracting the sarcoplasmic protein fraction from the *longissimus thoracis* muscle was performed as described by Costa et al. [21]. Initially, 100 mg of the muscle was homogenized using the Ultra-Turrax homogenizer (IKA T18 digital, Staufen, Germany) in 1 mL of lysis buffer composed of 20 mM Tris HCl pH 8, 5 mM EDTA, 1% protease inhibitor cocktail (Sigma-Aldrich®, San Luis, MO, USA), and 1% 2-mercaptoethanol (Sigma-Aldrich®, San Luis, MO, EUA), for 15 seconds. Subsequently, the homogenate was centrifuged (20.200 x g) for 20 minutes at 4°C. The supernatant (sarcoplasmic extract) was carefully collected, aliquoted, and stored at -80°C for subsequent analysis. The protein content was determined using the Bradford Protein Assay (Bio-Rad, Hercules, CA, USA).

Following the extraction and quantification process, 50 μg of the sample was placed into a tube, followed by the addition of 2.5 μL of 100 mM DTT (1.4-dithiothreitol; Sigma-Aldrich®, San Luis, MO, EUA). The mixture was then stirred and incubated in a heat block at 60°C for 30 minutes. Once the vial cooled to room temperature, 2.5 μL of 300 mM iodoacetamide was added to achieve cysteine alkylation. Due to its light sensitivity, the solution was vortexed and then kept in the dark at room temperature for 30 minutes. Subsequently, 10 μL of trypsin solution (Promega corporation, Madison, WI, USA) was mixed with ammonium bicarbonate, stirred, and incubated at 37°C overnight for protein digestion. Post-digestion process, the samples were then centrifuged at 18,000 x g at 6°C for 30 minutes, followed by drying in a Savant SpeedVac^TM^ concentrator (Thermo Fisher Scientific, Waltham, MA, USA).

The samples were reconstituted in 50 μL of 0.1% trifluoroacetic acid and underwent desalting using Zip-Tip according to the manufacturer’s guidelines. After the desalting process, the samples were dried again using a Savant SpeedVac™ concentrator and sent to the Analytical Center at IQ-USP (São Paulo, Brazil) for protein identification and quantification. A NanoAquity HPLC coupled with a maXis 3G high-resolution Q-TOF mass spectrometer (Bruker Daltonics, Billerica, MA, USA) was utilized for this analysis.

### 2.3. Data processing and protein identification

The mass spectrometer raw data were processed using the MaxQuant software (v1.6.3.3) with default settings, taking into account protein N-terminal acetylation, methionine oxidation as a variable modification, and cysteine carbamidomethylation as a fixed modification. The *Sus scrofa* reference proteome (ID: UP000008227), obtained from UniProt (www.uniprot.org, accessed on February 25, 2023), was used for protein identification. Trypsin specificity was maintained for the digestion mode, and the Bruker-QTOF instrument was set with default parameters, including peptide search tolerances of 20 ppm for the first search and 10 ppm for the main search. Label-free quantification (LFQ) was employed for quantification, with a false discovery rate (FDR) of 1% and a requirement for at least two unique peptides to ensure identification confidence.

### 2.4. Bioinformatics analyses

The Gene Ontology (GO) annotation, Kyoto Encyclopedia of Genes and Genomes (KEGG) pathway enrichment analysis, and protein-protein interaction (PPI) network analysis were conducted using String 11.0 software (accessed on February 6, 2024), with a confidence parameter of 0.4. PPI networks were generated for differentially abundant proteins (DAPs) between the CON and BET groups using the available interaction map of the *Sus scrofa* species. A significant enrichment of functional categorization in GO and KEGG pathways was determined using the Benjamini-Hochberg method, with an adjusted p-Value (*q*-Value) < 0.05 [27].

### 2.5. Intramuscular fat content and shear force measurement

The 3.0 cm thick *longissimus thoracis* samples from the carcass were used to determine IMF content and Warner-Bratzler shear force (WBSF), following the method described by Soares et al. [13]. Briefly, to evaluate IMF, a sampled chop was thawed and homogenized (TURRAX CT-132). IMF was measured using near-infrared spectroscopy (FoodScan, FOSS NIR Systems Inc., Laurel, MD). To determine WBSF, another chop was thawed and cooked in a water bath at 71°C for 40 minutes [13], and then cooled in a refrigerator overnight at 2–5°C before sampling for shear force measurement [28]. Six cylindrical cores (3.0 cm in height and 1.27 cm in diameter) were taken from each sample, cut parallel to the longitudinal orientation of the muscle fibers using a cork borer [28]. The WBSF was measured using a Warner-Bratzler Shear device (G-R Electrical Manufacturing Company, Manhattan, KS, USA). On this equipment, the cylindrical cores were cut in half through the center, perpendicular to the longitudinal orientation of the muscle fibers. A V-notch blade with a thickness of 1.016 mm and a 60° angle was used for this, operating at a constant speed of 20 cm/min. WBSF was expressed in Newtons (N).

### 2.6. Statistical analysis

The protein abundance data underwent quality control before analysis. Initially, 154 proteins were identified. However, to avoid issues with statistical analysis and inferences, proteins identified as potential contaminants, only identified by site and reverse sequence, were removed from the dataset, resulting in 120 proteins. Additionally, proteins represented in less than 15% of the samples, i.e., in 3 or fewer samples, were excluded [29]. The final dataset included 43 proteins. Under consideration in the statistical model were the fixed effects of groups (CON and BET) and statistical analysis was performed with the log2 transformed LFQ intensity values using the software Perseus (version 2.0.11) [30]. The comparison between groups was performed using a two-tailed independent Student’s t-test, with proteins considered differentially abundant (DAPs) when *q*-Value ≤ 0.05.

For the meat quality analyses (IMF and WBSF), the pig slaughtered at the end of the trial period was considered as the experimental unit. Assumptions of analysis of variance (ANOVA) were assessed, including normality of residuals using the Kolmogorov-Smirnov test and homogeneity using the Hartley’s F-maximum test. Statistical procedures were performed using R software (version 4.4.0; R Core Team 2024). The data were subjected to ANOVA, with statistical significance defined as a P-Value ≤ 0.05.

## 3. Results

### 3.1. Differentially abundant proteins

**Fig 1:**
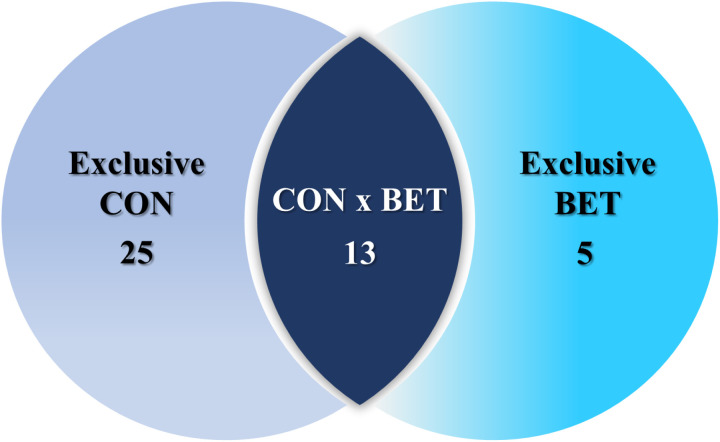
Venn diagram showing proteins unique to each diet group and those shared between them.

Following stringent protein selection based on pre-established criteria to ensure data quality, a total of 43 sarcoplasmic proteins were identified and compiled in the database originating from porcine *longissimus thoracis* muscle tissue. As depicted in the Venn diagram (Fig 1), 25 and 5 exclusive proteins were identified in the CON and BET groups, respectively. Furthermore, 13 proteins were found to be shared between the groups, which were subsequently investigated to identify DAPs. Among the proteins commonly identified in both groups, 11 DAPs showed significant differences (*q*-Value < 0.05), with all proteins being down-regulated in the BET group compared to the CON group (Table 1).

**Table 1:** Differentially abundant sarcoplasmic proteins (n = 11) identified between CON and BET groups.

Uniprot ID	Gene name	Full protein name	Fold Change (Log2)^1^	*q*-Value^2^
A0A286ZQE4	GAPDH	Glyceraldehyde-3-phosphate dehydrogenase	-0.71	0.004
A0A287AMK0	ALB	Serum albumin	-0.50	0.004
A0A286ZMZ9	PYGM	Alpha-1,4 glucan phosphorylase	-0.41	0.004
A0A287AQJ5	PKM	Multifunctional fusion protein	-0.51	0.006
A0A5G2R7R6	ENO3	Beta-enolase	-0.58	0.006
F1RNV1	GPI	Glucose-6-phosphate isomerase	-0.36	0.006
A0A4X1TS17	AGL	Glycogen debranching enzyme	-0.98	0.007
A0A288CFT0	TPI1	Triosephosphate isomerase	-0.36	0.029
A0A5G2R4C4	PGK1	Phosphoglycerate kinase 1	-0.41	0.031
A0A287A1V5	ALDOA	Fructose-bisphosphate aldolase	-0.42	0.037
A0A5G2QZN6	CKM	Creatine kinase M-type	-0.90	0.041

CON: control group; BET: betaine group; ^1^Negative fold change indicates the less abundant protein in the group BET compared to CON. ^2^*q*-Value < 0.05 indicates statistical significance.

“Exclusive CON” represents the number of proteins in the control group, “Exclusive BET” in the betaine group, and ‘CON x BET’ number of proteins common in both groups.

#### 3.1.1. Functional association analysis.

The results of GO enrichment of all DAPs for biological processes and molecular functions are presented in Table 2. In general, it was observed that all annotated biological processes (13 terms) and molecular functions (3 terms) were significantly down-regulated (q-Value < 0.05) following supplementation of pigs with BET. The results demonstrate that most of the DAPs were associated with carbohydrate metabolism, influencing biological processes such as: canonical glycolysis (*q*-Value = 0.003), glycolytic catabolic process (*q*-Value = 0.003), glycolytic process (*q*-Value = 1.41^-10^), cellular carbohydrate catabolic process (*q*-value = 0.00016), gluconeogenesis (*q*-Value = 0.00016), carbohydrate catabolic process (*q*-Value = 3.00^-13^), and glucose metabolic process (*q*-Value = 3.00^-5^). Within molecular functions, the processes most negatively affected by the supplementation of BET were intramolecular oxidoreductase activity, interconverting aldoses and ketoses (*q*-Value = 0.03), small molecule binding (*q*-Value = 0.04), and catalytic activity (*q*-Value = 0.03). Additionally, following the submission of DAPs to KEGG pathway enrichment analysis, similar findings were observed (Table 3). Nine pathways (*q*-Value < 0,05) were identified, with glycolysis/gluconeogenesis being the most significantly suppressed pathway, with seven proteins down-regulated in this pathway. Other pathways of significance also showed being affected, including: biosynthesis of amino acids, pentose phosphate pathway, fructose and mannose metabolism, carbon metabolism, starch and sucrose metabolism, HIF-1 signaling pathway, glucagon signaling pathway and metabolic pathways.

**Table 2:** Categorization of the functions of differentially abundant proteins (DAPs) according to biological processes and molecular functions.

GO-term	Description	*q*-Value^1^	Protein symbols^2^
*Biological process*			
GO:0061621	Canonical glycolysis	0.0033	PGK1, TPI1
GO:0005980	Glycogen catabolic process	0.0037	AGL, PYGM
GO:0006096	Glycolytic process	1.41^-10^	GAPDH, ENO3, PGK1, GPI, TPI1, ALDOA,
GO:0044275	Cellular carbohydrate catabolic process	0.00016	AGL, PYGM, TPI1
GO:0006094	Gluconeogenesis	0.00016	GPI, PGK1, TPI1
GO:0016052	Carbohydrate catabolic process	3.00^-13^	GAPDH, ENO3, PGK1, TPI1, PYGM, GPI, ALDOA, AGL
GO:0006006	Glucose metabolic process	3.00^-5^	GAPDH, PGK1, TPI1, GPI
GO:0019318	Hexose metabolic process	1.37^-6^	GAPDH, PGK1, TPI1, GPI, ALDOA
GO:0016051	Carbohydrate biosynthetic process	4.49^-5^	AGL, GPI, PGK1, TPI1
GO:0006091	Generation of precursor metabolites and energy	2.89^-10^	GAPDH, PGK1, TPI1, ENO3, PYGM, GPI, ALDOA, AGL
GO:0019637	Organophosphate metabolic process	6.80^-6^	GAPDH, PGK1, ENO3, GPI, ALDOA, CKM, TPI1
GO:0016310	Phosphorylation	1.87^-5^	GAPDH, PGK1, ENO3, TPI1, GPI, ALDOA, CKM
GO:0071704	Organic substance metabolic process	0.0332	GAPDH, ENO3, PGK1, TPI1, PKM, ALDOA, GPI, CKM
*Molecular function*			
GO:0016861	Intramolecular oxidoreductase activity, interconverting aldoses and ketoses	0.0332	GPI, TPI1
GO:0036094	Small molecule binding	0.0439	GAPDH, PGK1, ALDOA, ALB, GPI, PYGM, CKM
GO:0003824	Catalytic activity	0.0390	AGL, GPI, PGK1, PYGM, GAPDH, ALDOA, TPI1, ENO3, PKM, CKM

^1^The *q*-Value calculates according to the False Discovery Rate (FDR) method; ^2^Proteins corresponding to the connected proteins in the protein-protein interaction (PPI) network.

**Table 3:** Analysis of Kyoto Encyclopedia of Genes and Genomes (KEGG) of DAPs down-regulation in porcine skeletal muscle.

KEGG pathway	Description	*q*-Value^1^	Protein symbols^2^
ssc00010	Glycolysis/ Gluconeogenesis	1.52^-13^	GAPDH, ENO3, PGK1, TPI1, ALDOA, PKM, GPI
ssc01230	Biosynthesis of amino acids	4.25^-11^	GAPDH, ENO3, PGK1, TPI1, ALDOA, PKM
ssc00030	Pentose phosphate pathway	0.0043	GPI, ALDOA
ssc00051	Fructose and mannose metabolism	0.0043	ALDOA, TPI1
ssc01200	Carbon metabolism	4.06^-12^	GAPDH, ENO3, PGK1, TPI1, ALDOA, PKM, GPI
ssc00500	Starch and sucrose metabolism	0.0049	GPI, PYGM
ssc04066	HIF-1 signaling pathway	9.35^-6^	GAPDH, ENO3, PGK1, ALDOA
ssc04922	Glucagon signaling pathway	0.0325	PKM, PYGM
ssc01100	Metabolic pathways	3.51^-8^	GAPDH, ENO3, PGK1, TPI1, ALDOA, PKM, GPI, PYGM, CKM

^1^The *q*-Value calculates according to the False Discovery Rate (FDR) method; ^2^Proteins corresponding to the connected proteins in the protein-protein interaction (PPI) network.

#### 3.1.2. Protein-Protein Interaction (PPI) analysis.

**Fig 2:**
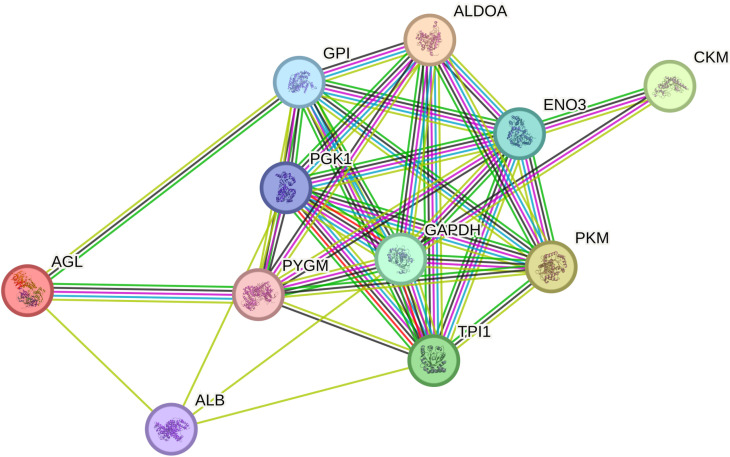
Protein-protein interaction network of differentially abundant proteins in *longissimus thoracis* muscle between CON and BET.

In most cases, certain biological processes and pathways may share the same set of proteins, suggesting a potential inter-linkage between them. The PPI of DAPs provides a deeper understanding of the key proteins affected by the supplementation of pigs with BET (Fig 2). The PPI network indicated that eight DAPs have multiple interactions and were identified as key nodes: glyceraldehyde-3-phosphate dehydrogenase (GAPDH), alpha-1,4-glucan phosphorylase (PYGM), multifunctional fusion protein (PKM), beta-enolase (ENO3), glucose-6-phosphate isomerase (GPI), triose phosphate isomerase (TPI1), phosphoglycerate kinase 1 (PGK1), and fructose-bisphosphate aldolase (ALDOA).

Nodes represent the differentially abundant proteins, and the lines represent the connection between proteins. PPI network analysis was done with Search Tool for the Retrieval of Interacting Genes/Proteins (STRING) website.

The analysis revealed a highly significant interaction network among the differentially abundant proteins (*p* < 1.0^-16^), indicating that down-regulation of these protein interactions may play a crucial role in modulating intracellular biological processes in pigs fed a BET diet.

### 3.2. Meat quality

**Fig 3:**
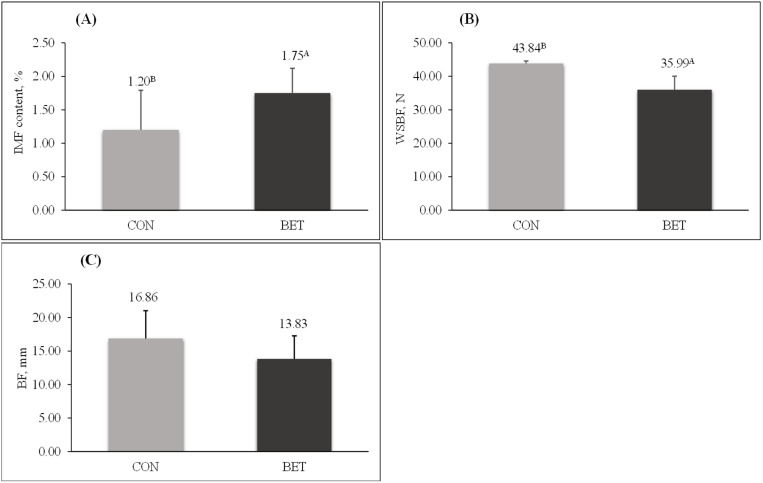
Intramuscular fat content, shear force measurements in *longissimus thoracis* muscle, and backfat thickness in betaine-supplemented pigs.

As shown in Fig 3, pigs fed the BET diet had higher (P = 0.02) IMF content in the *longissimus thoracis* muscle. Supporting this result, pigs in the BET group had lower (P < 0.01) WBSF compared to the CON group, indicating greater meat tenderness in pigs fed the BET diet. No differences (P = 0.111) were identified in the backfat thickness between pigs supplemented with BET and the CON group.

(A) Intramuscular fat content in control (CON) and betaine (BET) groups; (B) Warner Bratzler shear force in CON and BET groups; (C) Backfat thickness in pigs from the CON and BET groups. Means with different superscript letters indicate significant differences (p < 0.05) according to the F-test. Data previously published by Soares et al. [13].

## 4. Discussion

**Fig 4:**
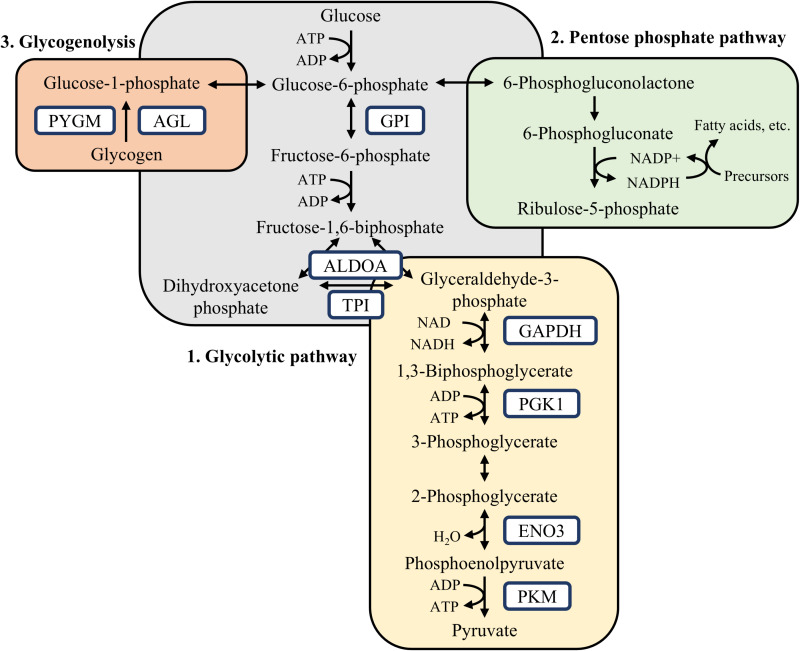
Summarized metabolic pathways influenced by DAPs.

According to the results of the GO annotation performed for the DAPs, the proteins GAPDH, ENO3, PGK1, GPI, TPI1, ALDOA are linked to carbohydrate metabolism. KEGG annotation and PPI analysis indicated that these down-regulated proteins are strongly associated with the glycolysis/gluconeogenesis pathway (Fig 4). Carbohydrate metabolism is a complex process that culminates in ATP formation, as well as the formation of essential metabolic intermediates in other molecular processes [31]. The reduction in glycolytic pathway activity and gluconeogenesis in pigs supplemented with BET suggests greater glucose availability in the organism to be utilized in other metabolic pathways, such as glycogenesis, *de novo* synthesis of fatty acids, and protein synthesis. Additionally, these results are consistent with the increased capacity for IMF deposition observed in this study, as well as the decline in post-mortem muscle pH, and the improvement in meat quality commonly observed in pigs supplemented with betaine during the finishing phase [4,5].

Glycolysis is depicted in gray and yellow, indicating the energy investment phase and the energy payoff phase, respectively. Glycogenolysis is shown in pink, and the pentose phosphate pathway is represented in green. Blue squares represent the DAPs, down-regulated in pigs supplemented with BET compared to the CON group. Adapted: Costa et al., [21].

Various cell types, including a small proportion of adipocytes, are naturally found in muscle tissue [32]. Carbohydrate metabolism regulates upstream *de novo* synthesis of fatty acids, influencing energy and lipid metabolism [17]. In this context, the results of this study suggest that betaine may modulate intramuscular lipid metabolism, as proteins related to glycolysis/gluconeogenesis such as TPI1, GPI, GAPDH, ENO3, PGK1 [16,33,34] are down-regulated in the skeletal muscle of animals with high IMF content, consistent with the phenotypic data of this study and from other research [3,12,14]. Through KEGG pathway analysis, it was revealed that the pentose phosphate pathway (PPP) is significantly associated with the DAPs. The down-regulated of these glycolytic enzymes may have a positive impact on the PPP, generating essential reducing compounds for *de novo* fatty acids synthesis, using glucose as the main carbon source [18,35].

The negative regulation of the GPI enzyme allows glucose-6-phosphate (G6P) to be diverted to the PPP, where G6P is metabolized to generate ribose-5-phosphate and, consequently, provides nicotinamide adenine dinucleotide phosphate (reduced form, NADPH), which acts as a cofactor in fatty acid synthesis, supplying the reducing groups necessary for biosynthesis reactions [18,36,37]. Furthermore, the inhibition of other glycolytic enzymes is correlated with increased PPP flux, such as GAPDH inactivation by chemical oxidation and feedback inhibition of the TPI1 enzyme [38,39,40].

The KEGG pathway analysis in this study also identified that the glucagon signaling pathway was altered in pigs fed the BET diet. Glucagon plays an important role in maintaining glucose homeostasis by increasing glycogenolysis and gluconeogenesis pathways [17]. Thus, betaine may indirectly act on the glucagon signaling pathway through the negative regulation of proteins associated with glycogenolysis (PYGM and AGL) and gluconeogenesis (GPI, PGK1, and TPI1). Furthermore, elevated levels of insulin and low levels of glucagon act as positive regulators of the enzyme glucose-6-phosphate dehydrogenase, a key enzyme in PPP regulation whose activity is higher in intramuscular adipose tissue compared to subcutaneous tissue [41,42]. Moreover, glucagon regulates the cytosolic enzyme acetyl-CoA carboxylase (ACC), which catalyzes the carboxylation of acetyl-CoA, resulting in the formation of malonyl-CoA, essential intermediate in fatty acid synthesis [43]. Although the predominant activity of this enzyme occurs in the hepatic tissue, evidence suggests that it is also present and active in skeletal muscle [14]. Glucagon applies its influence on ACC through phosphorylation, leading to enzyme inactivation and consequently reducing malonyl-CoA synthesis [43]. However, the activity of glucose-6-phosphate dehydrogenase and ACC was not directly assessed in this study, so its involvement in this metabolic process cannot be definitively concluded.

In our previously study [13] pigs fed BET exhibited increased mRNA expression in the PPARγ, C/EBPα, and FABP3 genes in muscle, compared to the CON group, reinforcing that betaine is capable of modulating lipid metabolism in muscle tissue, consistently with other observations [14,44]. PPARγ plays a crucial role in controlling adipogenesis, lipogenesis, and glucose homeostasis. Its activation promotes fatty acid esterification with glycerol, resulting in triglyceride synthesis in adipocytes and an increase in the size of these cells [45,46]. PPARγ also stimulates the expression of the transcription factor CCAAT/enhancer-binding protein alpha (C/EBPα), which, in turn, positively regulates the expression of PPARγ itself, ensuring the maintenance of adipocyte differentiation [45]. These transcription factors also influence the expression of genes such as FABP3, which plays an important role in the uptake and intracellular transport of long-chain fatty acids [47,48]. Thus, the results of this study highlight that the negative regulation of enzymes related to carbohydrate metabolism in pigs fed BET can significantly impact signaling pathways involved in the biosynthesis of compounds essential for *de novo* fatty acid synthesis. This effect, combined with the increase in expression of genes associated with lipogenesis in muscle tissue, suggests possible mechanisms underlying the increase in IMF in pigs supplemented with betaine and the consequent improvement in meat tenderness observed in this study.

Betaine supplementation did not cause changes in the performance of pigs compared to the CON group, but it did lead to a significant increase in loin eye area in the carcass, as previously reported by [13]. This effect may be partly related to the modulation of energy metabolism by betaine, as observed in the present study. Its zwitterionic nature, as described by [60], allows its accumulation in cellular organelles, where it can protect against osmotic and ionic stresses. This potentially reduces the energy expenditure required to maintain cellular homeostasis, thereby redirecting more energy toward anabolic processes, such as muscle growth [61]. Furthermore, [51] observed that betaine increases IRS1 (Insulin Receptor Substrate 1) phosphorylation, thereby activating the PKB (Protein Kinase B) signaling pathway, key molecular processes for protein synthesis and regulated by energy dynamics [13].

Another evidence of altered energy metabolism in pigs supplemented with betaine in this study was the negative regulation of the enzymes AGL and PYGM, associated with glycogenolysis, compared to the CON group. The enzyme PYGM is responsible for catalysis α-1,4-glycosidic bonds of glycogen in skeletal muscle, releasing glucose-1-phosphate [49]. However, it can only function until there are four glucose residues left in the branch before the α-1,6-branch point. To continue glycogen degradation beyond this point, AGL action is necessary. AGL transfers glucose units from the lateral string to the linear strand of glycogen, as well as hydrolyzes the branches, allowing PYGM to continue catalysis α-1,4-glycosidic bonds [50]. On the other hand, although no regulation of proteins associated with glycogen synthesis was observed in this study, betaine has been shown to stimulate glycogenesis, promoting glycogen synthesis through phosphorylation of the enzyme PKB, which inactivates the protein GSK3 (glycogen synthase kinase 3), responsible for inhibiting glycogen synthase activity [51,52]. Thus, due to the increased availability of glucose, together with the ability to stimulate synthesis and reduce degradation, the final balance may favor the accumulation of muscle glycogen in pigs fed BET diet.

These results are intriguing because betaine positively influences muscle deposition [51], and consequently, could affect glycogen stores. Such observations indicate a positive impact on glycogen accumulation in the muscles of pigs in the BET group. Larger glycogen reserves before slaughter exert beneficial influence on the extent of post-mortem pH decline [53]. The absence of glycogen interferes with the continuity of post-mortem glycolysis, resulting in higher final pH in pig meat, which directly impacts the typical texture and flavor properties of fresh meat [54,55]. The above mentioned is consistent with studies demonstrating that betaine did not impair the rate of pH decline [3,13,56] and improved the quality of pork [4,5].

Although the data from this study confirm the hypothesis that betaine supplementation modulates the sarcoplasmic proteome of finishing pigs, as evidenced by the increased IMF deposition in pigs fed the BET diet, we anticipated observing changes in the abundance of proteins directly involved in lipid metabolism. However, this effect was not detected. The absence of modifications in lipid-related proteins may be attributed to the limitations inherent in the bottom-up proteomic approach used in this study, which adopts the shotgun strategy [57]. Despite label-free quantification, with an FDR of 1% and the requirement of at least two unique peptides to ensure confidence in identification, being an effective approach for quantitative protein identification [21], it presents limitations in detecting post-translational modifications or alternative splicing variants [58]. Alternative splicing, for example, can generate protein isoforms that may not be correctly identified, as not all peptides of a given protein are necessarily measured or recognized, resulting in fragmented data [57,58]. Furthermore, many peptides are not unique, being shared by multiple proteins, which can lead to protein ambiguity groups, thereby complicating the accurate assignment of the present proteoforms [57,59]. Therefore, the absence of lipid metabolism-related proteins may, in part, be explained by these technical limitations, suggesting the need for more targeted approaches.

Overall, the results of the present study indicate that betaine supplementation in finishing pigs affects carbohydrate metabolism, down-regulating enzymes of the glycolytic and glycogenolytic pathways, and indirectly influences the glucagon signaling pathway. This suggests that excess available glucose may be redirected towards the formation of essential compounds for *de novo* fatty acid synthesis, as well as for the accumulation of muscle glycogen, thus influencing muscular energy metabolism and resulting in IMF accumulation. However, considering that betaine contributes to the synthesis of several metabolically active compounds [6,7], further studies are needed to verify whether the effect of betaine on glycolytic enzymes occurs directly or indirectly.

## 5. Conclusion

Betaine supplementation at 2.5 g/kg in finishing pigs has been shown to down-regulate proteins related to carbohydrate metabolism in skeletal muscle, including the suppression of glycolytic and gluconeogenic processes, as well as carbohydrate catabolic processes such as glycogenolysis. These results indicate a potential redistribution of glucose flow, possibly favoring other metabolic pathways, such as the pentose phosphate pathway. Furthermore, betaine supplementation enhances IMF deposition and consequently improves meat tenderness.
